# Is Active Moss Biomonitoring Comparable to Air Filter Standard Sampling?

**DOI:** 10.3390/ijerph19084706

**Published:** 2022-04-13

**Authors:** Paweł Świsłowski, Arkadiusz Nowak, Stanisław Wacławek, Zbigniew Ziembik, Małgorzata Rajfur

**Affiliations:** 1 Institute of Biology, University of Opole, Oleska St. 48, 45-022 Opole, Poland; anowak@uni.opole.pl; 2 Botanical Garden—Centre for Biodiversity Conservation, Polish Academy of Sciences, Prawdziwka St. 2, 02-973 Warsaw, Poland; 3 Institute for Nanomaterials, Advanced Technologies and Innovation, Technical University of Liberec, Studentská St. 1402/2, 461 17 Liberec, Czech Republic; stanislaw.waclawek@tul.cz; 4 Institute of Environmental Engineering and Biotechnology, University of Opole, B. Kominka St. 6a, 45-032 Opole, Poland; ziembik@uni.opole.pl (Z.Z.); rajfur@uni.opole.pl (M.R.)

**Keywords:** mosses, total suspended particulate (TSP), heavy metals, biomonitoring

## Abstract

Recently, significant attention has been paid to air quality awareness and its impact on human health, especially in urban agglomerations. Many types of dust samplers for air quality monitoring are used by governmental environmental monitoring agencies. However, these techniques are associated with high costs; as a consequence, biological methods such as active moss biomonitoring are being developed. The main disadvantages of such techniques are the lack of standardization of the preparation procedures and the lack of reliable comparisons of results with data from instrumental analyses. Our study aimed to compare the results obtained from active biomonitoring with the use of three moss species: *Pleurozium schreberi*, *Sphagnum fallax* and *Dicranum polysetum.* Samples were exposed via the moss-bag technique to measure the concentrations of analytes (Mn, Fe, Cu, Zn, Cd, Hg and Pb) which had accumulated among the total suspended particulates (TSP) collected from the filters of a dust collector in the city of Opole (Opole voivodeship, Poland). With regard to the physicochemical and biological traits of the mosses, their assessed lifetime and actual photochemical efficiency (yield) following exposure were meagre, which may have been related to the change of environment and their exposure to pollutants. When comparing the results obtained by the two methods used to monitor air pollution, the biomonitoring method was found to be incompletely consistent with the reference method. Biological monitoring using mosses must be carefully considered depending on the monitoring objectives, the required level of sensitivity and quality of measurement and the type of pollutant.

## 1. Introduction

Heavy metals in street dust originate from anthropogenic pollution [1]. This contamination contributes to air pollution and increasing concentrations of various fractions of particulate matter (PM) [2] as well as different levels of total suspended particles (TSP) [3,4]. Air pollution in urban areas leads to adverse health effects [5], so the scale of air quality research is increasing [6,7,8], leading to the growth and intensification of human biomonitoring [9,10,11].

In addition to classical air quality assessments and monitoring methods [12,13,14,15], other approaches are increasingly being used [16], with modeling, biota sampling and ecological indicators or green infrastructure being the most widespread [17,18,19,20]. One example is lichens [21] or mosses [22,23] for monitoring atmospheric aerosol quality. Plants are used extensively in environmental biomonitoring of PM pollution [24,25], and tree leaves have been employed in a national system for long-term biomonitoring of heavy metals in the air. The Romanian Ministry of Environment has implemented this system as a complementary tool to the National Air Quality Monitoring Network [26]. The same authors also incorporated the moss-bag technique into long-term monitoring of heavy metals in the air to further develop the BioMonRo monitoring tool [27]. In turn, the combined use of the moss-bag technique and emission inventories appears to be an effective approach for quantifying pollutants, and could be a part of a project to develop and improve air quality modelling [28].

In general, the number of studies in which biological methods are used to assess air pollution is increasing, but the proper preparation of biological materials and the measurement method should be taken into consideration [29]. Not many researches have undertaken direct comparisons between the results from active moss biomonitoring with those obtained from dust samplers [30] in order to integrate these methods in assessments of the viability of the aforementioned bioindicators [31]. This is compatible with the definition of biomonitoring and research in this field [32]. So far, comparisons have been made for passive biomonitoring of TSP [33,34]. Therefore, in this research, the challenge of comparing the results of active moss biomonitoring with instrumental measurements was addressed. TSP was chosen because dust of different fractions can be deposited on mosses [29,35].

In this work, for the first time to our knowledge, an attempt was made to correlate biomonitoring results with results from air monitoring. We have tried to verify the research hypothesis that concentrations of heavy metals accumulated in mosses are correlated to those in TSP dust deposited in filters. We expect to provide evidence supporting this hypothesis by several means, i.e., by: (I) evaluating metal concentration changes in TSP and mosses during exposition; (II) comparing TSP and elemental moss composition during exposition; (III) evaluating the relationships among metal concentrations; and (IV) controlling moss survival during exposure.

## 2. Materials and Methods

The moss species used for this study were *Pleurozium schreberi* (Pl), *Sphagnum fallax* (Sp) and *Dicranum polysetum* (Dp). They were collected in October 2020 from forests in the Swietokrzyskie Voivodship in southeastern Poland.

Moss samples were taken and prepared before exposure as part of active biomonitoring in accordance with the relevant guidelines [36]. According to a previously developed methodology, mosses were prepared before exposure [37]. Moss samples (27 bags, 3 g each) were hung on the viewing terrace of the building of the Institute of Environmental Engineering and Biotechnology of the University of Opole (Opole, PL). During the winter season, mosses were exposed for three months (27 October 2020–27 January 2021). After each month of exposure, three bags of each species were collected (1 month = 9 samples). At the same time, TSP were collected on QM-A quartz filters (Whatman, 47-mm diameter). The sampling time was 24 h, from noon to noon of the next day. TSP filters were changed every day for three months (i.e., a total of 81 filters). The airflow of the PNS3D15/LVS3D dust collector was 2.3 m^3^/h, in accordance with the standard procedure [38]. The concentrations of Mn, Fe, Cu, Zn, Cd, Hg and Pb in the filters before exposure were below the limit of quantification of the analytical method used.

After exposure, each moss sample, with a dry mass (d.m.) of 1.000 ± 0.001 g, and each filter were mineralized in a mixture of nitric acid and hydrogen peroxide using a Speedwave Four microwave oven (Berghof, DE) to determine the heavy metal contents. Anthropogenic emitters are the source of these analytes in the study area. The mineralization process was carried out at a temperature of 180 °C. For filters, this process was carried out at 220 °C, and was performed twice to ensure complete digestion of all dust samples, according to a method described in [39]. Heavy metals were quantified using an atomic absorption flame spectrometer type iCE 3500 (Thermo Scientific, Grand Island, NY, USA). Concentrations of metals were evaluated in solution after mineralization and filtration, and were diluted into volumetric flasks of 25 cm^3^. Calibration of the spectrometer was performed with standard solutions (ANALYTIKA Ltd., Prague, Czech Republic). The values of the highest concentrations of the models used for calibration (10 mg/dm^3^ for Fe, 7.5 mg/dm^3^ for Mn, 5 mg/dm^3^ for Cu, Zn, Pb, 2 mg/dm^3^ for Cd) were approved as linear limits to signal dependence on concentration. The concentration of Hg in the samples (0.04 g ± 0.001 g d.m.) was determined with an AMA 254 mercury analyzer (Altec Ltd., Prague, Czech Republic).

Table 1 presents the instrumental detection limits (*IDL*) and instrumental quantification limits (*IQL*) of the iCE 3500 spectrometer. Table 2 shows the concentrations of heavy metals in certified reference materials, i.e., BCR-482 *lichen*, produced at the Institute for Reference Materials and Measurements, Belgium.

The chlorophyll fluorescence of photosystem II and actual photochemical efficiency (yield) were measured using a modulated portable fluorometer (Opti-Sciences, Hudson, NH, USA) under ambient light conditions [42].

Comparisons of the metal concentrations in the mosses during the periods studied with the TSP sample composition were carried out in a multistep process. The first required adjustments of the time scales of the moss exposition and TSP collection. Since the moss samples had been exposed for one, two and three months, their compositions could not be compared with the metal contents in the daily TSP samples. To overcome this problem, the masses of the relevant components were calculated for each measurement day. Given the mass of the TSP sample and the metal concentrations, the mass of a given element was calculated. A monthly sum of TSP and metal masses was used for cumulated composition calculations.

The second problem was related to the incomparability of moss and TSP compositions. Besides the determined metals, both types of materials also contained many other components. The predominance of organic compounds in the mosses and mineral ones in TSP was expected. To compare the metal contents in mosses and TSP, the appropriate subcompositions were considered [43,44]. Concentrations *x_d_* in a *d* subcomposition could be derived from *D* compositions *x_D_* (*D* > *d*) using the formula:

(1) $$x_{d}=\frac{x_{D}}{\sum_{i=1}^{d}x_{D,i}}$$

where *x_D_* and *x_d_* are vectors of the concentrations, respectively, in terms of composition and subcomposition. The components used in subcomposition formation were numbered from 1 to *d*.

A problem related to numerous data being below the detection limit occurred. However, individual observations lower than the detection limit (*BDL*) would not affect conclusions resulting from the data elaboration. Nevertheless, plentiful *BDL*s might have significantly affected the sum of the calculated metal abundances. To overcome this problem, data imputation for *BDL*s was applied. For this purpose, computations were conducted in R language version 4.1.0 [45]; the *multLN* function in the R language and the *zCompositions* library were used [46].

**Figure 1:**
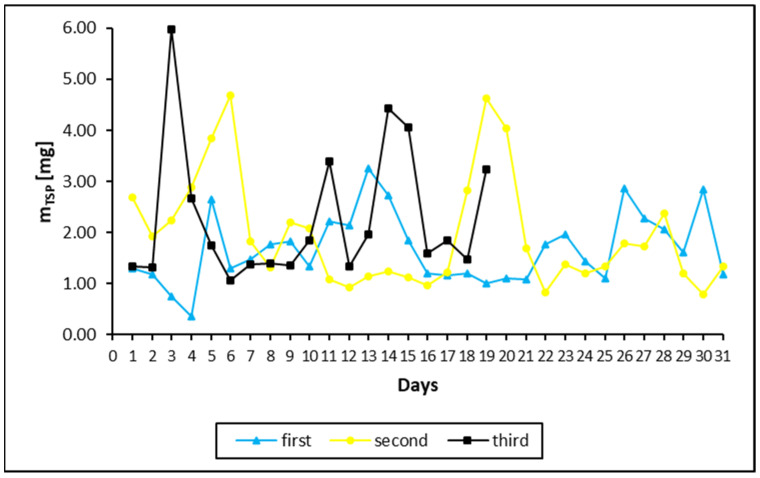
Daily TSP mass changes during the first (blue triangles), second (yellow dots) and third months (black squares) of sample collections in filters. The differences in the duration and daily sampling of the TSP filters were due to breaks associated with the Christmas and New Year holidays and a technical fault with the dust collector pump lasting 14 days.

The third problem arose from the discontinuity of the data collection. The TSP collection was corrupted by incidental breaks in sample collections (Figure 1). To supplement the data, a temporary liner trend was considered. Since temporal changes in the metal concentrations were low during the period studied, linear interpolation was sufficient to describe concentration changes over time. The missing data for breaks were calculated using the estimated course of concentration changes throughout the sampling period.

The contents of elements Mn, Fe, Cu, Zn, Cd, Hg, and Pb in subcomposition (*i* = {1,..., 7}) for TSP and moss were calculated. In this way, metal concentrations in the material could be determined independently of other component abundances, facilitating comparisons of the results from TSP and moss.

Despite different materials and collection periods, the procedures applied in the data elaboration process enabled the comparison of moss and TSP compositions. As a result, valid conclusions about the material compositions could be drawn.

## 3. Results

The daily mass increments of TSP in the filters are shown in Figure 1. As shown, there was considerable variation in the amount of deposited dust over time, and there were differences from one month to another. The average daily weight of TSP intake was 0.0016, 0.0020 and 0.0023 g for the first, second and third months, respectively.

The variable daily amount of dust deposited on the filters was also reflected in the concentrations of heavy metals detected therein, as shown in Table 3.

In general, the order of element abundances in TSP samples was: Fe > Pb > Zn > Cu > Mn > Hg > Cd for first month; Fe > Zn > Pb > Cu > Mn > Cd > Hg for the second; and Fe > Zn > Pb > Cu > Mn > Hg > Cd for the third.

**Figure 2:**
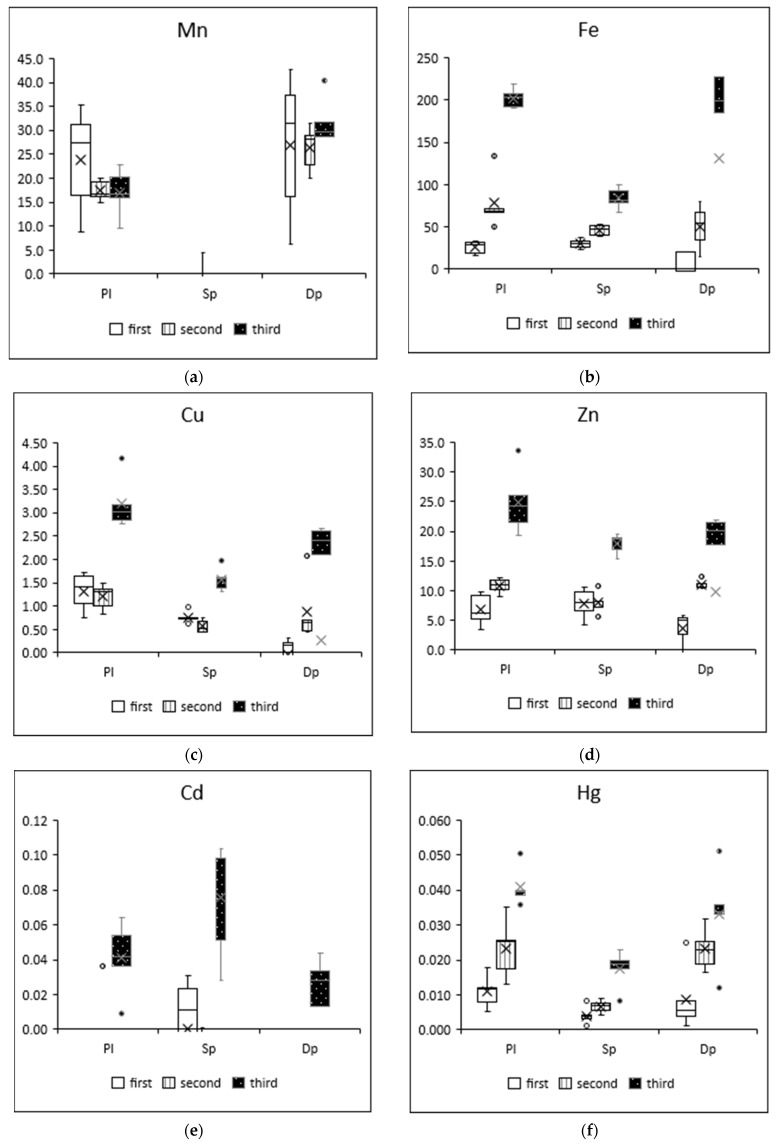

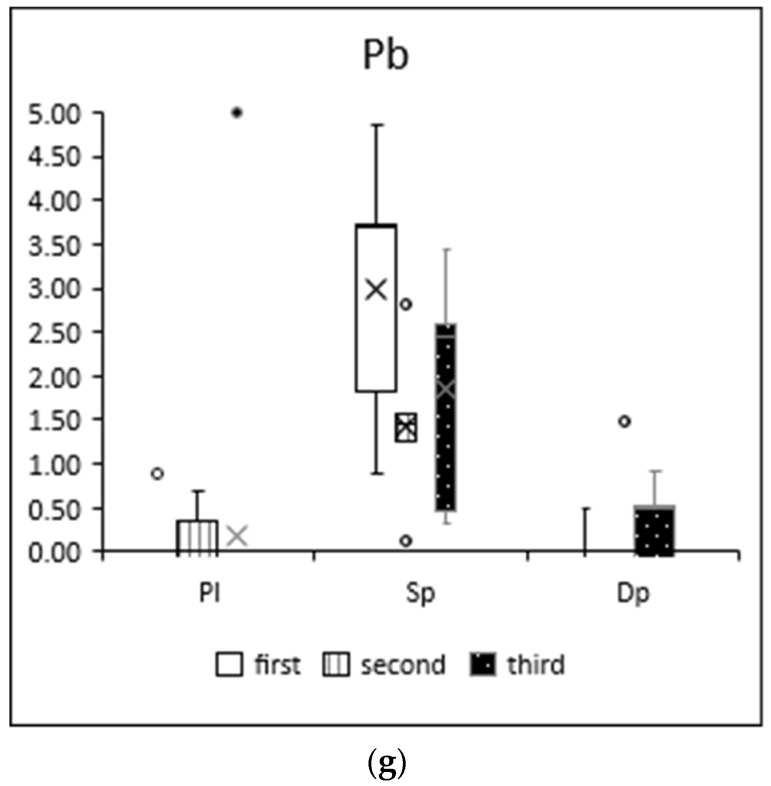
Elemental concentrations (mg/kg d.m.) of (**a**) manganese, (**b**) iron, (**c**) copper, (**d**) zinc, (**e**) cadmium, (**f**) mercury and (**g**) lead in the mosses after the first (white), second (white with stripes) and third (black with dots) month of exposure. Elemental concentrations determined in mosses prior to exposure are presented in Supplementary Materials, Table S1.

The results of the moss-bag technique showed increases in heavy metal concentrations in three moss species depending on exposure time and element, as shown in Figure 2. The values shown are the increases, i.e., the relative concentrations, which are the differences between the concentrations measured in the moss after exposure (C_af_) and those in the control sample before exposure (C_be_): (C_af_ − C_be_).

The results shown in Figure 2 indicate changes in element concentrations over time. Increases in these metals can be observed from month to month; for most elements and moss species, the greatest increases in concentrations were observed after three months of exposure (iron, copper, zinc, cadmium, mercury).

A linear model was constructed to describe changes in metal concentrations with respect to exposition duration and moss species. The model is described by the symbolic expression [47]:

log(*c*) ~ (*moss species*) ∗ (*exposition duration*) (2)

The values of structural parameters β_i_, their standard errors *SE*_β_, and p-values for the null hypothesis H_0,i_: β_pop,i_ = 0 (β_pop,i_ is the *i*-th (*i* = {0,1}) structural parameter in the general data population) were calculated. For the β_0_ and β_1_ parameters and differences β_{0,1}_({Pl,Sp} − β_{0,1}_(Dp)), the 95 % confidence intervals *CI* (in the range from 2.5% to 97.5%) were calculated. The results are presented in Table S2 in the Supplementary Materials.

The computation results led to the following conclusions. For Mn, Fe and Hg, statistically significant differences in the starting concentrations described by β_0_ in the moss species were found. In comparison to *D. polysetum*, the Mn concentration was higher in *P. schreberi* and lower in *S. fallax*. In *P. schreberi* and *S. fallax*, the Fe and Hg concentrations were lower than in *D. polysetum*. For Cd and Pb, the differences between β_0_ parameters for *D. polysetum*–*P. schreberi* and *D. polysetum*–*S. fallax* were statistically insignificant. The difference in Cu concentration for *D. polysetum* and *P. schreberi* was statistically insignificant, but the concentration in *S. fallax* was significantly lower than that in *D. polysetum*. A similar effect as that for Cu was observed for Zn, but the concentration in *S. fallax* was higher than in *P. schreberi* and *D. polysetum*.

Changes in the metal concentration over time are described by slope β_1_. Concerning moss species, β_1_ indicates an increase in metal concentration during exposition (β_1,pop_ > 0) or no changes (β_1,pop_ = 0). No rinse effect on the metal concentration (β_1,pop_ < 0) was noticed. No statistically significant changes in Mn and Pb concentrations during the study period in the moss species were observed. The increase in Cu, Cd and Hg concentrations was not related to the moss species. The difference in the accumulation rate of Fe and Zn in *D. polysetum* and *P. schreberi* was statistically insignificant. In *S. fallax*, a smaller increase in metal concentrations was observed.

**Figure 3:**
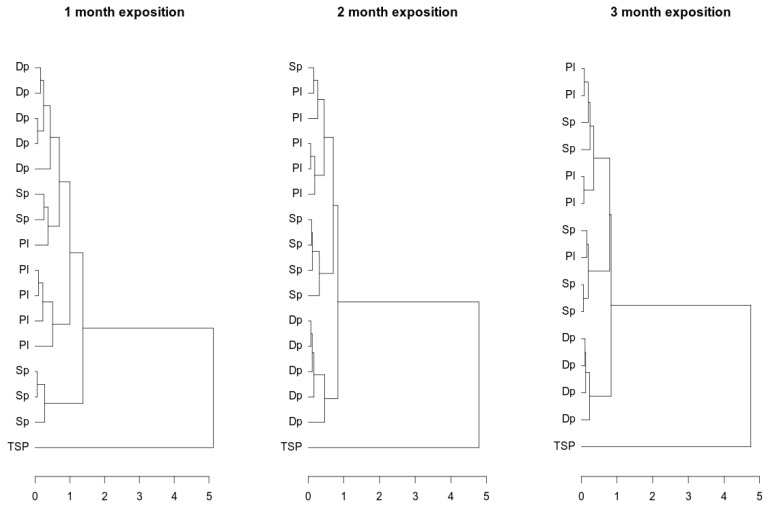
Cluster analysis of heavy metals in three moss species and in filter TSP.

The composition of metals accumulated in mosses and filters was compared in the next step. For the subcomposition comprising Mn, Fe, Cu, Zn, and Pb, [43] the distances between points representing the metal contents in the moss samples and TSP were calculated. In the dendrogram (Figure 3), the structure of the distances is shown. To determine the structure of the clusters, a complete linkage method was used. Two main clusters representing moss and TSP were observed. Within the moss cluster, three subclusters can be recognized. One of them presents *D. polysetum*, independent of the exposure period. The remaining clusters represent the other moss species, which were not uniquely assigned to groups.

The proportionality of metal concentrations in the materials was studied. To assess concentration covariabilty variance, logarithmized concentration ratio *t* was calculated. The *t* parameter was calculated with the formula:

(3) $$t_{jl}=\mathrm{var}\left(ln\frac{c_{j}}{c_{l}}\right)$$

The value of the *t* parameter was low, revealing a common trend in concentration changes, i.e., an increase in c*_j_* was followed by an increase in c_l_. To estimate the co-variability in the concentration of the metal pairs, *t*_jl_ (*j*,*l* = {Mn, Fe, Cu, Zn, Pb}) were calculated according to a method described in [48]. The results are presented in Table S3 in the Supplementary Materials. For Fe and Pb (first month of exposition), a low *t*_jl_ value indicating concentration change tendency was observed. For Pb and Zn concentrations in the second and third months of exposition, a similar trend was observed. The standard increase in Pb and Zn concentrations could be assigned to low emissions during the heating season [49,50].

**Figure 4:**
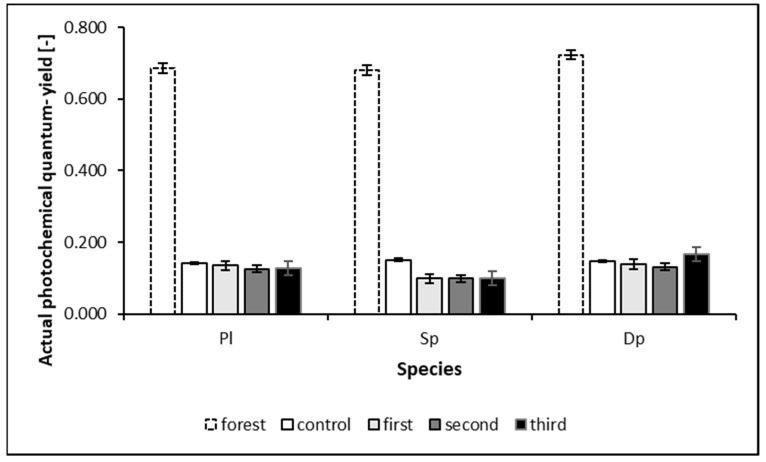
Changes in actual photochemical quantum yield (PQY) with duration of exposure (whiskers indicate a standard deviation).

The influence of heavy metal pollution and environmental conditions causes significant variability in the lifespan of mosses, as shown in Figure 4.

The graph in Figure 4 indicates that all species are susceptible to environmental change. Their vitality decreases dramatically when they experience unfavorable variable meteorological-environmental conditions and air pollution in autumn and winter. During the experiment, the exposed mosses significantly decreased their photosystem II activity. The rate of accumulation of elements depends on environmental conditions [51]; in the same way, the result of a photosynthetic activity measurement is dependent on the conditions under which it is performed [52]. Sudden changes in environmental conditions and (associated with it) stress cause the moss condition to deteriorate [53,54].

A multiple regression model was used to assess the effect of metal concentrations and exposure time on the life span, as measured by POY (Table 3 SM). In this model, the POY variable describes a specific metal concentration and exposure time. Calculations were performed separately for each of the moss species studied. The results indicated that the viability of the mosses decreased over successive months of exposure. This was indicated by the negative value of the structural parameter describing changes in POY during successive exposure periods (*p* < 0.05 is marked in yellow in Table 3 SM). With exposure time, viability decreased for *D. polysetum* in terms of iron concentration. For *S. fallax*, this trend was observed with Mn, Cd and Pb. For *P. schreberi*, on the other hand, viability decreased over time in terms of Fe, Cu, Zn, Cd and Hg concentrations. When considering separately the effect of changes in element concentrations over time after the calculated coefficient of variation (mean of three months), the results indicated that low concentration variation generally did not affect moss viability. Concentration variability higher than 10% resulted in increased viability with increasing iron concentrations for *D. polysetum* and increasing lead and mercury concentrations for *P. schreberi*.

## 4. Discussion

The sorption of pollutants by mosses has already been discussed many times [55,56], but it is essential to take into account the mechanism of this process, especially when considering active forms and bioaccumulation [57,58,59,60,61,62]. However, depending on the testing method, their context and purpose must be taken into account [63]. The elemental concentrations shown in Figure 2 indicate a cumulative trend over time. This observation was consistent with previous literature studies, in which the concentrations of some elements (Fe, Zn, Cd and Hg) in *Sphagnum girgensohnii* increased continuously (linear accumulation trend) with exposure time [64]. The concentration of elements in mosses was influenced by the time of exposure; during the heating season, it was observed that moss samples were particularly enriched in Cu or Zn [65]. The moss-bag technique with *Sphagnum junghuhnianum* also confirmed higher concentrations of elements in winter than in summer; additionally, for Cu, Pb and Zn, it showed that the source was traffic pollution [66]. The same sources can be attributed to the mosses exposed in this study. In the city center during the winter, combined with the previously mentioned low emissions, an effect was noted on the enrichment of heavy metals in mosses [50].

In our opinion, moss vitality measurements should not be excluded during experiments [31]. Despite the high proportion of analyses using devitalized mosses [67,68,69,70], we still believe that this approach is inadequate, according to the definition of biomonitoring and bioindicators, i.e., living indicator organisms [71,72,73,74]. Otherwise, we treat moss only as a chemical adsorbent, i.e., a natural sorbent [75,76,77] that has nothing to do with biomonitoring.

Despite significant damage to moss tissue and cell integrity after exposure, they are able to efficiently accumulate airborne trace elements [78]. However, heavy metal concentrations were not a determinant factor of moss vitality (Table S4) [79]. Suitable sample preparation prior to exposure homogenizes the sample material, as indicated by the low variability in metal concentrations (*CV*) [37]. In most cases, the small percentage of variation in the concentrations of metallic elements accumulated by the moss (less than 10%) did not adversely affect its lifespan. In contrast, Pb and Hg values higher than those observed (10%) represented a positive change in the vitality of *P. schreberi*, as did Fe values for *D. polysetum*. This supports the conclusion that elemental concentrations in moss after exposure are independent of the vitality of the organism [80]. Most elements (Fe, Cu, Zn, Cd and Hg) showed a cumulative trend in the moss with the length of exposure for the three species. Time of exposure (together with the accompanying variable environmental conditions) negatively affected the vitality of the mosses according to our analyses [81]. We recommended using a single species to monitor atmospheric pollution because different species of mosses have different accumulation capacities [82]. We found *D. polysetum* to have the highest accumulation capacity for regional monitoring of the atmosphere [83], but our study did not confirm this. In the case of our study, *P. schreberi*, which is used for active and passive biomonitoring studies, proved to be the best [84,85]. Other metals did not show such a trend; this was attributed to the influence of precipitation, which may have washed the metals away [86] (among other factors). The monthly rainfall was 35.6 mm, 18.6 mm and 37.4 mm respectively, for the studied exposure months [87]. In previous studies, we have shown how important it is to consider the influence of environmental conditions on the accumulation of heavy metals by mosses [88,89].

The anatomical and structural features of the plant influence which and how much PM they capture [90,91]. Although mosses capture mainly fine particles (<2.5 µm), the results from Pseudoscleropodium *purum* moss indicated that particles entrapped by mosses represent different fractions, and the amount of PM was strongly related to the concentration of metallic elements [92]. Other studies have confirmed that *Hypnum* *cupressiforme* entraps a prevalence of potentially inhalable or breathable particles (≤PM10), where the smallest particle classes were predominant [93]. The need to compare biomonitoring results with other methods is also stressed for TSP [35,94]. Hence, in our study, we decided to investigate dust in the whole TSP fraction, and not only selected PM.

In the first case, a comparison of biomonitoring studies with an automatic device using *H. cupressiforme* mosses and cellulose filters yielded different results: cellulose filters showed the lowest accumulation ability [95]. The interception and accumulation of airborne particles in exposed moss bags occur through different mechanisms than those involved in the PM10 collection by automatic devices [96]. A correlation analysis between PM10 API (Air Pollution Index) in the air and depositions of S, Pb, Cu, Zn in the moss bags showed a significant correlation with the concentration of Cu [74]. In contrast, in an Austrian experiment, toxic elements in mosses correlated well with data on overall air pollution obtained by the Index of Atmospheric Purity (IAP) method [97]. Other studies in the field of passive biomonitoring have indicated that bryophytes are suitable for the verification of air pollution in mathematical models of PM10 due to their ability to capture the long-term deposition of pollutants [98]. In another case, it was shown that this moss bag technique (using *S girgensohnii*) could be a valuable tool to verify model performance; both methods showed the same trend [99]. However, in most of the works cited, studies referred to PM10 in the dust as well as in moss [30]. We find it particularly difficult to understand how, for the latter case, heavy metals were quantified only in PM10 in relation to mosses. The authors concluded that there was no statistically significant difference between the two methods (*S. girgensohnii* moss bags and PM10 samples); however, we cannot find any statistical analysis confirming this [30].

The examples cited above indicate that different fractions of dust (mainly fine) are deposited in plants. Comparing elemental concentrations in PM10 deposited on filters with elemental concentrations in mosses where there are different fractions yielded inconsistent results in terms of pollutants from different fractions. From our point of view, we think this is the wrong approach, so we decided to collect TSP in filters (and quantify the heavy metals therein), and we treated mosses the same way (they also collected different fractions, including TSP). In the future, more attention should be paid to research on the dust fractions that are deposited in mosses (depending on the species); only then should they be compared to the corresponding PM fractions deposited on filters (this applies to both biomonitoring methods). Therefore, biomonitoring studies should be compared in-house, with considerable attention being paid to how contamination affects the viability of the bioindicator. This method shows the form of accumulation of contaminants (in our case, heavy metals) and their effects on mosses. We recommend continuing research into this phenomenon and standardizing further procedures associated with the moss-bag technique.

## 5. Conclusions

In our study, we tested three moss species, i.e., *P. schreberi*, *S. fallax* and *D. polysetum*, with the objective of verifying the hypothesis that the concentrations of heavy metals accumulated in mosses are proportional to those in TSP dust deposited in the filter.

We found that the most abundant elemental components in the collected TSP particles were Fe, Pb and Zn, whereas lower concentrations of Mn, Hg and Cd were detected.

Concentration changes over time were related to the moss species and the element in question. Excluding Cd and Pb, the initial metal concentrations were related to the moss species.

No prevailing rinse phenomenon was observed. No statistically significant changes in Mn and Pb concentrations by moss species were observed. An increase in Cu, Cd, and Hg concentrations was not related to the moss species. In *S. fallax*, a smaller increase in the metal concentrations was revealed.

Moss species are sensitive to environmental changes. Their vitality decreased when exposed to unfavorable meteorological and environmental conditions or air pollution. Despite the significantly decreased activity in photosystem II, the exposed moss was still able to accumulate TSP components from its surroundings.

The clusters observed in dendrograms for moss composition were distinct from TSP composition clusters. This observation led us to conclude that the elemental compositions of moss and TSP are significantly different. One factor influencing the biological activity of the moss is its affinity for chemical compounds; this brought about differences between TSP and moss composition.

A common trend in terms of changes in Pb and Zn concentrations indicated low emission sources as the main origin of these metals in the TSP.

The present research indicates that the results obtained by the two methods (active biomonitoring and deposited dust on the filter) have different applications. Mosses accumulate bioavailable forms of metals and are affected by many external factors during exposure (thus changing their degree of vitality); therefore, the results were different from those obtained with an automatic device.

## Figures and Tables

**Table 1:** The instrumental detection limits (*IDL*) and instrumental quantification limits (*IQL*) of the iCE 3500 (mg/L) spectrometer [40].

Metal	*IDL*	*IQL*
**Mn**	0.0016	0.020
**Fe**	0.0043	0.050
**Cu**	0.0045	0.033
**Zn**	0.0033	0.010
**Cd**	0.0028	0.013
**Pb**	0.0130	0.070

**Table 2:** Comparison of measured and certified concentrations in BCR-482 *lichen* [41].

	BCR-482 *lichen*		AAS (n = 5)		*Dev.* **
Metal	Concentration	Measurement Uncertainty	Average	±*SD* * of the Concentrations	
	[mg/kg d.m.]				[%]
**Mn**	33.0	0.50	31.7	0.68	−3.90
**Fe**	804	160	771	154	−4.10
**Cu**	7.03	0.19	6.63	0.17	−5.70
**Zn**	100.6	2.20	95.1	2.30	−5.50
**Cd**	0.56	0.02	0.53	0.03	−5.30
**Pb**	40.9	1.40	38.2	1.00	−6.60

* standard deviation. ** relative difference between the measured (c_m_) and certified (c_c_) concentration 100% (c_m_ − c_c_)/c_c_.

**Table 3:** Monthly element abundances (ng m^−3^) detected in TSP filter samples.

	Mn	Fe	Cu	Zn	Cd	Hg	Pb
1st month min	64.1	6305	202	951	21.7	67.5	124
max	473	29,664	586	4433	21.7	88.8	6049
median	182	11,036	327	2091	21.7	81.6	2312
average	189	12,033	352	2215	21.7	79.3	2216
*SD*	81.8	4613	115	804	-	10.8	1442
n	31	31	31	31	1	3	31
2nd month min	93.5	6335	182	1066	8.70	49.2	443
max	661	29,712	584	6545	207	69.2	5499
median	213	10,138	320	2240	117	68.7	1892
average	252	11,163	357	2634	103	62.4	2065
*SD*	117	4801	112	1454	57.8	11.4	1159
n	31	31	31	31	31	3	31
3rd month min	8.70	3774	196	691	4.35	74.5	234
max	247	19,722	834	7184	59.8	81.0	8963
median	96.7	7575	358	1245	35.3	78.5	1011
average	110	8953	432	2453	34.4	78.0	1651
*SD*	82.7	3855	183	2003	20.4	3.29	2049
n	15	19	19	19	8	3	19

## Data Availability

Not applicable.

## Supplementary Materials

The following supporting information can be downloaded at: https://www.mdpi.com/article/10.3390/ijerph19084706/s1, Table S1: Mean metal concentration in mosses before exposure (mg/kg d.m.),Table S2: Structural parameters (Estimate) of the linear model describing changes in element (Element) concentration in plant in relation to time of exposure, Table S3: Estimates of co-variability type in pairs of elements’ concentrations during periods of expositions, Table S4: Structural parameters (Estimate) of the linear model describing changes in photochemical quantum yield (POY) in relation to element’s concentration and the plant species (Species).
