# Supplementary material for: Is Active Moss Biomonitoring Comparable to Air Filter Standard Sampling?
Source: Int J Environ Res Public Health. 2022 Apr 13;19(8):4706. doi: 10.3390/ijerph19084706 (PMC9024558; doi:10.3390/ijerph19084706)
Supplement: Supplementary file 1 [file ijerph-19-04706-s001.zip › Table S1.pdf]

Table S1. Mean metal concentration in mosses before exposure (mg/kg d.m.).

	**Mn**	**Fe**	**Cu**	**Zn**	**Cd**	**Hg**	**Pb**
Pl	212	173	7.07	52.2	0.51	0.031	6.65
Sp	151	128	3.59	30.1	0.53	0.036	2.97
Dp	168	306	8.49	32.8	0.54	0.049	7.41
n	5	5	5	5	5	5	5
